# Freeze-Drying of Platelet-Rich Plasma: The Quest for Standardization

**DOI:** 10.3390/ijms21186904

**Published:** 2020-09-20

**Authors:** Isabel Andia, Arantza Perez-Valle, Cristina Del Amo, Nicola Maffulli

**Affiliations:** 1 Bioprinting Laboratory, Regenerative Therapies, Biocruces Bizkaia Health Research Institute, Cruces University Hospital, Plaza Cruces 12, 48903 Barakaldo, Bizkaia, Spain; arantza.perezvalle@osakidetza.eus (A.P.-V.); cristina.delamomateos@osakidetza.eus (C.D.A.); 2 Department of Musculoskeletal Disorders, University of Salerno School of Medicine and Dentristry, 84084 Salerno, Italy; n.maffulli@qmul.ac.uk; 3 Queen Mary University of London, Barts and the London School of Medicine and Dentistry, London E1 4DG, UK

**Keywords:** platelet-rich plasma, fibrin, freeze-drying, wound healing, orthopedics, dentistry

## Abstract

The complex biology of platelets and their involvement in tissue repair and inflammation have inspired the development of platelet-rich plasma (PRP) therapies for a broad array of medical needs. However, clinical advances are hampered by the fact that PRP products, doses and treatment protocols are far from being standardized. Freeze-drying PRP (FD-PRP) preserves platelet function, cytokine concentration and functionality, and has been proposed as a consistent method for product standardization and fabrication of an off-the-shelf product with improved stability and readiness for future uses. Here, we present the current state of experimental and clinical FD-PRP research in the different medical areas in which PRP has potential to meet prevailing medical needs. A systematic search, according to PRISMA (Preferred Reported Items for Systematic Reviews and Meta-Analyses) guidelines, showed that research is mostly focused on wound healing, i.e., developing combination products for ulcer management. Injectable hydrogels are investigated for lumbar fusion and knee conditions. In dentistry, combination products permit slow kinetics of growth factor release and functionalized membranes for guided bone regeneration.

## 1. Introduction

The complex biology of platelets and their involvement in inflammation and tissue repair have inspired the development of platelet-rich plasma (PRP) therapies for a broad array of medical needs, expanding from wound healing and dental applications to musculoskeletal medicine, ophthalmology, dermal conditions, gynecology and urology, among others [1].

The clinical benefits of PRP or platelet-rich fibrin (PRF) (MeSH terms since 2007 and 2018, individually) are being investigated in more than 575 and 222 registered clinical trials, respectively (clinicaltrials.gov, accessed 11 August 2020). Furthermore, thousands of research articles have been published in the last decade on different aspects of PRP science, including investigations on the pleiotropic functions of platelet secretome in nonhemostatic events.

Generally, PRP is used fresh and in an autologous fashion. The absence of concerns about PRP safety has fueled its application in unmet medical conditions, i.e., conditions whose treatment are unsatisfactory with current available therapies, including but not limited to osteoarthritis and chronic wounds. However, clinical advances are hampered by the fact that PRP products, doses and treatment protocols are far from being standardized [2]. Additional drawbacks come from an operational perspective, such as the requirement of qualified staff for blood manipulation and medical facilities complying with regulatory requirements to ensure sterility and safety.

In this context, PRP lyophilization, i.e., freezing followed by water sublimation and subsequent removal of water vapor, has been proposed as a consistent method for product standardization and fabrication of an off-the-shelf product with improved stability, ready for future uses. The lyophilization of blood-derived products has been widely studied. Massive transfusion of freeze-dried plasma (FDP) was initiated during the Second World War to overcome logistic problems, such as the cooling and management of bags with high-plasma volumes that often cracked [3,4]. Despite good results in containing traumatic hemorrhages in the military field and catastrophic settings, its allogenic use was interrupted because of disease transmission (i.e., hepatitis, HIV). At present, however, safety can be ensured by using infection screening on donations, leucodepletion or virus inactivation among others. Only a few countries, including Germany, Norway and Denmark, use it for civilian care [3], and, current research is insufficient to encourage the use of FDP [5].

In transfusion science, maintaining platelet integrity and function is paramount to fulfill their hemostatic function [6]. Thus, the quality of FD-PRP is determined by platelet integrity and response to agonists, as well as the activity of coagulation factors, ability to produce thrombin, and clot strength after rehydration [7]. Instead, PRP science is grounded in the interactions of platelet’s secretome with injured tissues (other than vasculature). Indeed, functional proteins released from the a-granules in the platelets are associated with angiogenesis, cell growth and adhesion, inflammation and cell metabolism [8]. Therefore, research to demonstrate equivalence to fresh PRP is mainly based on the assessment of the concentration levels of cytokines that are relevant in healing mechanisms, and the demonstration of the maintenance of their biological activity by in vitro and/or in vivo functional assays.

Lyophilized or freeze-dried PRP (FD-PRP) can be prepared on an autologous basis, which is convenient for treatments requiring multiple applications. In addition, allogeneic FD-PRP from single or pooled donors can be safely prepared using blood from healthy people. FD-PRP can be stored at room temperature for several months, reconstituted easily and used on demand. This review presents the current state of experimental and clinical FD-PRP research in the different medical areas in which PRP has potential to meet current medical needs.

## 2. Published Articles on Freeze-Dried PRP

**Figure 1:**
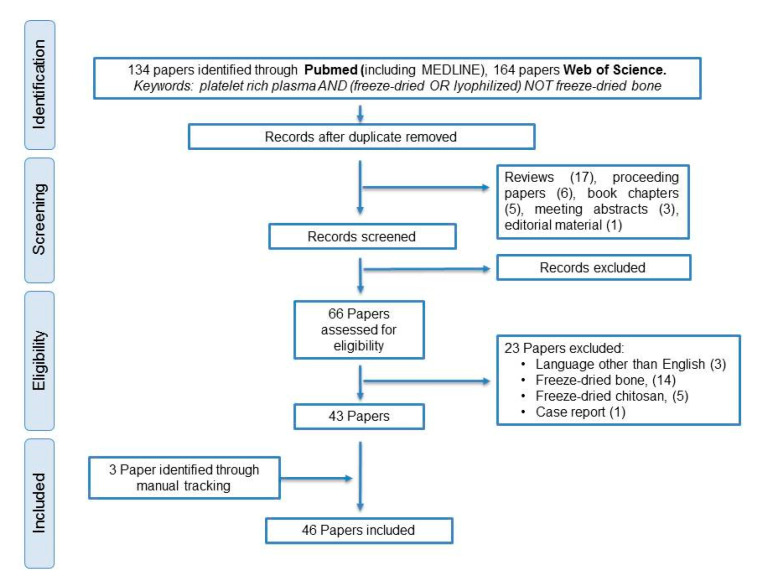
PRISMA (Preferred Reported Items for Systematic Reviews and Meta-Analyses) workflow.

We performed a systematic search, according to PRISMA guidelines from inception until 15 July 2020, using the following data bases: Web of Science (WOS) and PubMed (including MEDLINE) (Figure 1). The following search strategy was used: platelet-rich plasma and (freeze-dried or lyophilized) not freeze-dried bone. Original articles were included if they reported the use of freeze-dried platelet-rich plasma (FD-PRP) or fibrin (FD-PRF) in either experimental or clinical research in applications other than transfusion. Reviews, proceedings, meeting abstracts, book chapters, editorial material and case reports were excluded. Publications were reviewed by at least two researchers, and included articles grouped according to medical field application or those assessing FD-PRP preservation. Of the 46 included articles, skin research was addressed in 17 papers, mainly addressing wound management (15 papers). Other areas of interest were musculoskeletal conditions (10 studies), mainly addressing osteoarticular problems and dentistry (7 studies). Another study examined the feasibility of eye drop preservation for ocular surface conditions [9].

### 2.1. Lyophilized PRP Preservation

Platelet-rich plasma preparations: relevant aspects for FD-PRP formulations (resting vs. activated platelets).

Freeze-drying PRP products are encouraged to secure the preservation and standardization of PRP therapies, and thereby boost the clinical benefits that they provide. Quality control of the process is mandatory to guarantee the maintenance of the biological activity of the signaling proteins within each formulation separately.

**Figure 2:**
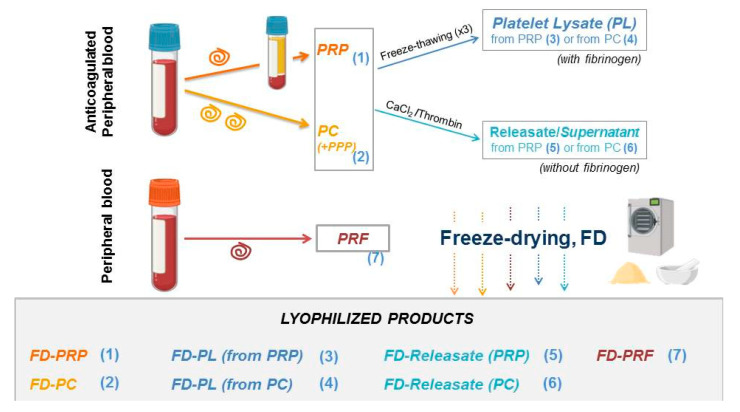
Preparation of different plasma formulations for lyophilization. Single spinning of anticoagulated whole blood yields platelet-rich plasma (PRP) with moderate concentration of platelets (and optionally leukocytes), for the purpose of this study designed as (1); alternatively, double spinning or plateletpheresis systems produce platelet concentrates (PCs) designed as (2). The pellet can be resuspended in a determined volume of Platelet-Poor Plasma (PPP) and platelet count adjusted, or platelets can be used without plasma. When PRP or PC products are freeze-thawed multiple times (commonly 3) or sonicated, the resultant platelet lysates (PLs) contains platelet secretome along with platelet membranes (ghost platelets) designed as (3) and (4), respectively. Alternatively, the platelet secretome can be obtained after the activation of platelets by adding calcium chloride/ thrombin. The supernatant released from the clot (from PRP (5) or PCs (6)) contains the whole platelet secretome. PRF (7) is obtained by centrifuging noncoagulated peripheral blood and disposing of the formed hydrogel the red blood cells. Terminology: PRP, platelet-rich plasma; PC, platelet concentrate; PPP, platelet-poor plasma; PL, platelet lysate; PRF, platelet-rich fibrin; FD-PRP, freeze-dried platelet-rich plasma; FD-PC, freeze-dried platelet concentrate; FD-PL, freeze-dried platelet lysate; FD-PRF, freeze-dried platelet-rich fibrin.

Figure 2 depicts the different PRP formulations that have been lyophilized thus far. For preservation (equivalence) studies, they can be gathered in two groups: (A) products that preserve platelet integrity, (products (1), (2) and (7)), and (B) lysates and releasates mostly composed of platelet secretome (from either PRP or platelet concentrates (PCs), designed as (3)–(6) in Figure 2).

In lyophilized products from group (A), the platelet membrane should be undamaged after the freeze-drying process [7]: changes in size distribution, altered morphology by SEM, biomarker pattern in flow cytometry, and response to agonists after platelet rehydration are therefore assessed. In this context, platelets can be preserved by introducing trehalose, a commonly used lyoprotectant, into their cytosol, and freeze-drying in trehalose/albumin buffer [10]. Trehalose is also efficient as an extracellular lyoprotectant, preventing protein aggregation and enhancing plasma stability during storage, and is preferred over sucrose and glucose [11].

The comparison of the kinetics of the Growth Factor (GF) release of calcium-activated fresh PRP and calcium-activated rehydrated freeze-dried platelet-rich plasma (FD-PRP) treated with trehalose evidenced differences in the kinetics of platelet-derived growth factor (PDGF) and transforming growth factor (TGF-b) release but not vascular endothelial growth factor (VEGF) [12]. Therefore, Pan et al. suggest that these products should be Ca^+2^-activated prior to freeze-drying to avoid these differences.

In lyophilized products from group (B), the concentration of alpha-granule proteins are often measured as an index of platelet secretome. In addition, functional assays in vitro are performed to assess signaling proteins’ activities. The rationale for the clinical use of PRP is mainly based on the administration of platelet secretome, particularly the protein pool stored in alpha-granules; each platelet contains approximately 50–80 alpha-granules that release their content upon platelet activation [13]. Instead of the previously identified 300 proteins in a-granules [14,15], new proteomic data revealed that, after collagen and thrombin stimulation of platelets, only 124 proteins constitute the platelet releasate. The discrepancy in protein number is attributed to the subtraction of proteins present in resting platelets [16]. Concentrations differ among proteins, varying from highly abundant (ug/mL), i.e., thrombospondin, PF-4 and von Willebrand factor, to less abundant (pg/mL), such as VEGF. The crucial paradox of platelets is that they contain proteins with opposing functional roles; for example, PF-4 is antiangiogenic while VEGF stimulates endothelial cell proliferation.

Despite containing proteins from different functional categories, most studies focus on the growth factor family (GF). Typically, PDGF, TGF, epidermal growth factor (EGF) and VEGF are involved in tissue repair and assessed in fresh versus FD-PRP. For example, Shiga et al. [17] compared fresh-frozen (−80 ºC) PRP with FD-PRP. They established its stability, up to 8 weeks at room temperature, based on the quantification of PDGF, VEGF, TGF and EGF. Similarly, da Silva et al. [18] verified the stability of PDGF, VEGF, TGF and EGF comparing fresh PRP and FD-PRP (double spinning protocol) and proved the activity by measuring human umbilical endothelial cells (HUVEC) and fibroblast proliferation.

Pooling the PRP of different patients has emerged as a safe option for the standardization of these products. To validate this methodology, Kieb et al. [19] quantified GFs, in particular VEGF, β-FGF, PDGF-AB, TGF-β1, IGF-1, IL-1α, IL-1β and IL-1RA in peripheral blood, fresh PRP prepared using a commercial system, fresh-frozen irradiated PC, and irradiated FD-PC. The different preparations had similar levels of GFs overall, although IGF-1 and IL-1α could not be detected in FD-PCs, since they are plasma proteins, not platelet proteins.

In addition, the safety of FD-PRP is paramount and has to be demonstrated to regulatory bodies by donor testing before donation and one month later to ensure that no virus incubation has escaped from analyses. Sterility can be achieved by gamma irradiation of the extracted product without affecting the efficacy of FD-PL in chondrocytes, fibroblast, osteoblasts and mesenchymal stem cell (BM-MSC) cultures [20]. Indeed, supplementing cultures with 5% FD-PL showed advantages vs. 10% FBS in terms of proliferation, especially at low cell density. These results support the use of human-derived supplements in vitro, moving into a more biomimetic model and avoiding the use of xenogeneic derivatives.

### 2.2. Skin research

#### 2.2.1. Wound Management

Chronic nonhealing wounds produce a heavy economic and social burden. Their management requires a multimodal approach depending on their etiology. For example, compression therapy is crucial in venous leg ulcers, while adequate foot off-loading is critical in diabetic foot ulcers. Regardless of the etiology of the ulcers, there is consensus regarding the need to optimize the biological conditions of the ulcer bed and its microenvironment. PRPs can provide advantages by augmenting the concepts behind the TIME framework for ulcer care (tissue management, inflammation and infection control, moisture balance and edge (epithelial) advancement) [21]. Indeed, PRPs can help in tissue management by providing a pool of signaling proteins, which can trigger healing mechanisms [22].

Ulcer care requires multiple PRP applications, normally entailing several venipunctures in patients with multimorbidity. In such patients, FD-PRP (either autologous or allogenic), would offer tangible benefits, since levels and biological activities of PRP are conserved through the lyophilization process [23,24] (Table 1).

Many chronic wounds are colonized with bacteria hampering the healing process. The inhibitory actions of PRPs on the growth of various pathogenic bacteria strains is platelet dose-dependent, with platelet-poor plasma showing no impact on bacterial growth [25,26]. Actually, platelets express receptors members of the Toll-like receptor (TLR) family, which bind to bacterial targets, thereby leading to platelet activation and the release of microbicidal proteins and cytokines contained within platelets, including thrombocidins from CTAP-III and NAP-2, kinocidins, beta defensing -1, -2, -3 and thymosin-beta 4 [27]. These proteins exert their role through the recruitment of circulating neutrophils involved in bacterial clearance. Other specific platelet membrane receptors also bind to Escherichia coli or staphylococcal proteins, facilitating neutrophil entry and the debridement of the wound site [28].

In addition, the antibacterial activities of PRP can be improved by optimizing the configuration of combination products. Wang et al. [29] fabricated an antibacterial against *S. aureus*, *P. aeruginosa* and *C. albicans* composite dressing using chitosan/silk fibroin nanosilver loaded with PRP (prepared through double spinning), and assessed the physical and mechanical properties of the device, protein release, biological safety (silver content in organs) and efficacy in infectious wound treatment (CTS-SF/Ag/SA/PRP) in vitro and in vivo. In wound healing studies, Yassin et al. [30] compared PRP wafers (carboxymethyl cellulose, CMC) and powder and antibacterial activities against Gram-negative bacteria. FD-wafers were proposed as PRP delivery systems: they showed better results than PRP powder in a rat wound model, in terms of antimicrobial efficacy, wound size measurements and histopathological analyses. The designed product was proven stable for 3 months at −20 °C.

Moisture balance is beneficial to ensure appropriate wound care and can be achieved by combining PRP with biomaterials commonly used for this purpose in the manufacture of wound dressings. Combination products obtained through the mixture of FD-PRP with alginate [31], CMC [30] or gelatin [32] facilitate moisture preservation in the wound bed while serving as vehicles for sustained release, to match with the biological requirements of the wound [31].

Collagen is commonly used in wound dressings, and Horimizu et al. [33] designed collagen sponges as vehicles for FD-PRP with positive results in a diabetic mice wound model [33]. In another example, different concentrations of FD-platelet lysates were encapsulated in collagen, and the equivalence between fresh and lyophilized products was shown by scratch wound assays, chicken chorioallantoic membrane (CAM) assays, degradation assessment and PDGF-BB and VEGF release [34]. The combination of FD-PRP with collagen products derived from the porcine extracellular matrix (so-called ADM, acellular dermal matrix) promoted neovascularization, collagen deposition and epithelialization [24]. Moreover, FD-PRP can also be combined with other biomaterials. The mechanical and physicochemical properties of silk cocoon combined with FD-PRP offer an advanced wound dressing, which enhanced wound closure in New Zealand rabbits [35].

The use of PRP in specific wound etiologies, such as diabetic foot, is based on high quality clinical evidence [36], but clinical data reporting the efficacy of FD-PRP are limited. FD-PRP powder (1 × 10^7^ platelets/cm^2^) was used in the management of deep second degree burn wounds in the plantar area showing significant beneficial differences in the healing rate at 3 weeks and bacteria colonization compared to controls [37].

#### 2.2.2. Other Conditions

The high prevalence of androgenetic alopecia and the limited efficacy of current available treatments have prompted the clinical investigation with PRP. A recent meta-analysis has shown that PRP can improve hair density and thickness [38]. Usually, several sessions of microneedling with PRP are needed, often associated to minoxidil [39]. In an initial industrial-scale approach, the lyophilization of activated PRP from pigs was effective in the expansion of human follicle dermal papilla cells. Thus, this can yield an off-the-shelf product with potential for alopecia management [40]. Striae distensae is a skin condition especially common in females, causing cosmetic morbidity and psychological distress. Augmenting fractional CO_2_ ablational laser procedures with lyophilized growth factors has been shown to improve clinical outcomes [41]. 

**Table 1:** Skin research.

Author, Year, (Reference)	Condition	FD-PRP Based Product/Stability	Study Type/Cells/Animal Model	Results
**Wound healing**				
Horimizu M 2013 [33]	Wounds	Lyophilized collagen sponge coated with PRP Stability at 4 °C: at least 3 months	Human periosteal fibroblasts Diabetic mice model GF antibody microarray Biomechanical characterization Histology: vessel formation, cell number, presence of adipose tissue, steatosis	Stimulation of cell growth in vitro Enhanced wound healing and regenerative potential in vivo
Huber CS 2019 [23]	Acute Wounds	Saline vs. fresh PRP vs. FD-PRP	Wistar male rats GF release assessments Histology: coll. deposition, Masson’s trichrome, Wound closure kinetics	EGF, PDGF-AA, TGF-b1 and VEGF levels are conserved in FD-PRP No differences in epithelial thickness, collagen density and wound closure kinetics Enhanced presence of myofibroblasts and vascularization with FD-PRP
Lei X 2019 [24]	Acute Full-thickness wounds	Porcine ADM+FD-PRP vs. fresh PRP vs. ADM vs. control	C57 Mouse model Healing evaluation: inflammation, vascularization, epithelialization, collagen deposition	TGF-b1, EGF, PDGF-AA, VEGF levels in ADM+FD-PRP were lower than in PRP Wound closure enhanced with FD-PRP/ADM: it promotes wound healing, neovascularization, collagen deposition and epithelialization
Lima AC 2014 [34]	Wounds	FD-PL encapsulated in collagen, hASCs encapsulated in coll+PL beads Fresh PL vs. FD-PL	GF release Beads degradation hASCs activities Chick CAM assay	No changes in VEGF, PDGF-BB release over 72h; sustained GF release. No differences in hASCs proliferation, scratch wound assay and angiogenesis
Liu J 2017 [35]	Wounds	FD-(Silk cocoon+PRP) vs. FD-(Silk cocoon+PPP) vs. Mepitel	L929 cell activites Wounds closure in the back of New Zealand white rabbits	FD-(Silk cocoon+PRP) enhanced L929 proliferation and wound
Nardini M 2020 [31]	Full-thickness chronic wounds	Alginate/SS vs. FD-PL/Alg/SS vs. Alg/FD-PL	GF release kinetics hBMMSCs, hFB: Cell viability, proliferation and oxidative stress. Western blot: cyclin D1 Mouse model C57/BL6: granulation tissue, early inflammation, collagen deposition, fibroblasts maturation, re-epithelialization, neovascularization	Enhanced GF release over 144h from FD-PL/Alg/SS compared to FD-PL/Alg Increased proliferation and Cylcin D1 expression in FDPL FD-PL/SS rescued the cells from oxidative stress and supported cell proliferation Faster wound healing results with FD-PL/Alg/SS in vivo
Notodihardjo SC 2018 [42]	Full-thickness wounds	(PL vs. CL-PL vs. FD-PL vs. FBS) + gelatin Stability at 4 °C: 9 months	GF release hFBs bioactivity Histology in mice: wound area, neovascularization, granulation tissue formation	The levels of PDGF-BB, VEGF and TGF-b1 were reduced in FD conditions Bioactivity of FD is maintained: increased hFBs proliferation in PL conditions vs. FBS No differences in wound healing in vivo
Notodihardjo SC 2019 [32]	Full-thickness wounds	FD-PL vs. different concentrations of FD-PL + gelatin Stability at 4 °C: 9 months	C57BL6J/Jcl mice Histology: H&E, Azan and anti-CD31	Gelatin sheets impregnated with 2- and 3-fold FD-PL concentrations accelerated the healing process by favoring the formation of granulation tissue and capillaries in vivo
Pietramaggiori G 2006 [43]	Dorsal Wounds (Diabetic)	FD-PRP, 1.2 × 10^6^ plts/ul vs. fresh-frozen PRP vs. sonicated PRP	Diabetic mouse model Assessment of GFs Histology: cell proliferation, angiogenesis, wound thickness, surface coverage	No differences in PDGF, TGF-b, EGF and VEGF concentrations: preservation maintained Increased tissue formation with FD-PRP and fresh-frozen PRP
Pietramaggiori G 2008 [44]	Wounds (Diabetic)	ADM vs. FD-PRP vs. ADM-FD-PRP	Diabetic mouse model FBs Wound healing kinetics and new tissue formation	The combination of ADM-FD-PRP stimulate fibroblasts proliferation in vitro and revascularization and tissue formation in vivo
Sell SA 2012 [45]	Wounds	FD-PRP vs. MH vs. MH-FD-PRP	hFBs, macrophages and endothelial cell activities	FD-PRP and MH-FD-PRP conditions enhance cell activities: proliferation, collagen matrix deposition and migration
Wang Q 2019 [29]	Wounds	Chitosan/silk fibroin nanosilver loaded with FD-PRP	BALBc mice Physical and mechanical properties Protein release Biological safety (silver content in organs) Antibacterial properties	Good asymmetric performance, appropriate physical and mechanical properties, slow release of proteins. Wound moisture retention and promotion of healing.
Xu F 2018 [46]	Acute full-thickness dorsal skin wounds	Different concentrations of FD-PRF on a PVA hydrogel	Cell activities in L929 and HUVECs Wound healing histologic assesment in mice.	1% of FD-PRF-PVA hydrogels: - Accelerated wound closure - Enhanced granulation tissue, maturity, collagen deposition and capillary formation
Yassin GE 2019 [30]	Wounds	FD-PRP + CMC (wafers) vs. FD-PRP powder Stability at −20 °C: 3 months	Rat wound model Antibacterial activities against Gram-negative bacteria	FD-PRP wafers present greater antimicrobial efficacy and wound size reduction
Yeung CY 2018 [37]	Deep second degree burn wounds in the plantar area	FD-PRP (dose: 1 × 10^7^ platelets/cm^2^), vs. conventional care	Clinical study	Significant reduction in the wound healing rate and bacterial colonization
**Other Dermal Applications**				
Abdallah M 2020 [41]	Striae distensae (SD)	FD-GF vs. CO_2_ ablational laser, and combination of both methods	Clinical trial, 20 female patients. Each patient, 3 therapy methods Before treatment and six weeks after: - Assessment of clinical score (reduction % of SD width, appearance, color, size) - Histopathologic examination	The combination of ablational laser and FD-GF was clinically more effective than ablational laser alone
Lin YK 2016 [40]	Hair	FBS vs. FD-porcine PRP vs. fresh porcine PRP	hFDPCs activities GF release: elisa, MTT, PCR	Higher GF levels in PRP than FBS and it is stable. No difference in HFDPCs activities in fresh or FD-PRP and FBS

Abbreviatures: ADM, acellular dermal matrix; Alg, alginate; CL-PL, cryopreserved platelet lysate; CMC, carboxymethyl cellulose; coll, collagen; FBS, fetal bovine serum; FD, freeze-dried; FD-PL, freeze-dried platelet lysate; FD-PRP, freeze-dried platelet-rich plasma; GF, growth factor; H&E, hematoxylin and eosin; hASCs, human adipose-derived stem cells; hBMMSCs, human bone marrow mesenchymal stem cells; hFBs, human fibroblasts; hFDPCs, human follicle dermal papilla cells; MH, Manuka Honey; PC, platelet concentrate; PDL, periodontal ligament; PL, platelet lysate; PPP, platelet-poor plasma; PRP, platelet-rich plasma; PVA, polyvinyl alcohol; SD, Striae distensae; SS, silk sericrin.

## 3. Musculoskeletal Applications

Osteoarthritis (OA) is a prevalent degenerative joint condition with no effective treatments available other than surgery. An unsustainable economic burden by 2030 has been predicted due to the continuous growth of joint replacements [47]. Identifying conservative treatments for earlier disease stages is paramount. A recent meta-analysis [48] including 26 randomized studies showed that intra-articular injections of PRP were more effective than hyaluronic acid in terms of pain reduction and functional improvement, with comparable safety profiles. Therefore, FD-PRP can offer important advantages in this context, as most often PRP treatments for knee OA involve multiple injections at each treatment cycle. Preliminary clinical data showed significant improvement in pain, function and quality of life 1, 3 and 6 months after injection of autologous PRP powder resuspended in 6 mL of normosaline [49] (Table 2). Beneficial clinical effects are attributed to the modulation of inflammation.

A common in vitro functional model consists of exposing chondrocytes or cartilage explants to IL-1β and measuring how PRP interferes with the inflammatory response triggered by IL-1β. In this framework, Jain et al. [50] showed that the encapsulation of FD-PRP in polyethylene glycol enhanced PRP effects by rescuing chondrocyte proliferation and decreasing MMP13-induced extracellular matrix catabolism. The latter was achieved through a reduction in common inflammatory molecular pathways, such as NF-kB activation and nitric oxide (NO) synthesis. Similarly, dose-related chondroprotective actions of PRP, without differences between FD-PRP compared to fresh-frozen PRP, were found in equine cartilage cultures [51]. In a similar way, standardized human PRP powder induced a dose- and time-response relationship in human chondrocyte proliferation and metabolic activity [52].

A promising approach is the combination of PRP with biomaterials such as tissue scaffolds. In this way, alginate was functionalized with FD-PRP by creating amide bonds with PRP peptides through carbodiimide chemistry [53] and compared to PRP encapsulated in alginate. The resulting injectable hydrogels were studied in nucleus pulposus cells and FD-PRP-functionalized alginate showed higher levels of cell survival and increased secretion of glycosaminoglycans (GAGs ) over time, compared to encapsulated PRP.

In a rat model of spinal posterolateral fusion, bone union was accelerated by FD-PRP at a comparable rate to fresh PRP and performed better than BMPs (Bone Morphogenetic Proteins). In this study, all were combined with an artificial bone substitute, hydroxyapatite/collagen [54]. In addition, these authors confirmed the pharmacological activity of FD-PRP after a four-week storage by examining the PDGFR/ERK signal transduction in osteoblasts [55].

Tendon and ligament injuries are particularly relevant in sports medicine and orthopedics [56]. Clinical data on the effects of PRP in commonly injured tendons including Achilles and patellar tendons, common extensors and rotator cuffs have been published, but meta-analyses are not conclusive, in part given the lack of PRP standardization. Pioneering studies using commercial trehalose-treated lyophilized platelets in equine research models similar gene activation than fresh-frozen PRP in tendon cell cultures [57]. Furthermore, FD-PRP is an effective carrier for icariin, and accelerated tendon–bone healing in New Zealand rabbits with partial patellectomy, as assessed by histology and mechanical testing [58]. Additionally, the use of polydioxanone fibers containing FD-PRP in volumetric muscle loss caused a dose- and ERK-dependent effect in myogenic differentiation [59]. Recently, the feasibility of dry storage of allogeneic FD-PRP has been illustrated in the treatment of plantar fasciopathy [60].

**Table 2:** Studies in musculoskeletal research.

Author, Year (Reference)	Condition	FD-PRP Based Product/Stability	Study Type/Cells/Animal Model	Results	
**Musculoskeletal Pathology**					
Camargo-Martin L 2019 [51]	Equine OA	Frozen-PRP vs. FD-PRP vs. filtered FD-PRP 1.5-, 3- and 6-fold platelet enrichment	Equine cartilage explants exposed to PRP, FBS and ITS as controls	Better chondroprotective effects with 3-fold PRP products compared to controls No differences between FD-PRP and frozen PRP	
Growney EA 2020 [53]	Spine	FD-(PRP biofunctionalized alginate) vs. FD-(PRP encapsulated alginate) vs. alginate control Double spinned PRP	hNPCs viability, adhesion and ECM and GAG secretion in hypoxic and normoxic conditions	Decreased cytotoxicity in the presence of PRP Increased hNPCs adhesion and distribution in PRP-functionalized alginate No differences in cell proliferation Increased GAG content and ECM production in PRP-functionalized alginate in hypoxic conditions	
Hahn O 2020 [52]	Cartilage conditions	FD-PRP vs. PRP powder Different PRP stimulation frequency and doses	Chondrocyte cultures for 14 days	Pro-collagen type 1 and -3, GAGs and cell proliferation were time-dependent and increased with FD-PRP concentration	
Jain E 2019 [50]	OA	Double spin PRP, comparison of bolus PRP vs. FD-PRP encapsulated in PEG	Kinetics of VEGF, EGF, PDGF-BB and TGF-b1 release until degradation of hydrogel IL-1b treated chondrocytes	VEGF and EGF are released on day 1 while TGF-b and PDGF-BB present a sustained release PRP rescued cell proliferation No effect on NO synthesis Does not rescue changes in gene expression induced by IL-1b Bolus PRP decreased inflammatory NF-kB activation	
Kinoshita H 2020 [55]	Spine	Fresh PRP vs. FD-PRP Stability: PRP 4 weeks	Osteoblast proliferation and ERK and PDGFR phosphorilation	FD-PRP is functionally (phosphorylation mechanisms) equivalent to fresh PRP	
Shiga Y 2016 [54]	Lumbar fusion	FD-PRP (thrombin, CaCl_2_-activated) + artificial bone vs. fresh PRP + artificial bone vs. BMP + artificial bone vs. autologous bone	Spinal posterolateral fusion in rats Radiography and histology: amount of bone formation, characteristics of trabecular bone Biomechanical strength (3-point bending test) PDGF and TGFb1 determinations	(FD-PRP + artificial bone) accelerated bone union at a rate comparable to (fresh PRP + artificial bone) or (BMP + artificial bone) More trabecular branches and biomechanical rigidity at 8 weeks	
Shirata T 2019 [49]	OA	FD-PRP Stability: 6 months	Clinical study Intraarticular injection of FD-PRP (resuspended in normosaline)	Enhanced clinical outcomes (KOOS score) 1, 3, 6 months post treatment	
**Tendon and Muscle**					
McCarrell T 2009 [57]	Tendon and ligament	BMA vs. PRP vs. FD-PRP	Flexor digitorum superficialis tendon and suspensory ligament explants: TGF-b1 and PDGF release RT-QPCR: COL1A1, COL3A1, COMP, MMP-3 MMP-13	TGF-b1 and PDGF concentrations higher in PRPs than BMA Correlation between GF concentrations and ECM gene expression PRP and FD-PRP better outcome than BMA Platelet concentration correlated with ECM gene expression	
McClure MJ 2018 [59]	Volumetric muscle loss	Aligned electrospun polydioxanone vs. random oriented, loaded with FD-PRP powder	C2C12 murine myoblasts: cell morphology, cell signaling multiplex assay, myogenic gene expression and protein and integrin synthesis and in response to FD-PRP	Compared to random scaffold, fiber alignment + FD-PRP powder favors myogenic differentiation, which is ERK-dependent and dose-dependent	
Zheng C 2019 [58]	Tendon bone interface	PRP (double spin, Ca^+2^-activated) mixed with ICA and lyophilized vs. FD-PRP vs. control	New Zealand rabbits, partial patellectomy At 8 weeks and 16 weeks, microcomputed tomography, histology, biomechanical testing	Sustained release of ICA from FD-PRP+ICA compared to fresh PRP Higher rate of bone formation and remodeling in FD-PRP+ICA Better new bone formation in FD-PRP+ICA Fibrocartilage zone formation in the three groups, better mechanical properties in FD-PRP+ICA	

Abbreviatures: BMA, bone marrow aspirate; BMP, bone morphogenetic protein; ECM, extracellular matrix; FBS, fetal bovine serum; FD-PRP, freeze-dried platelet-rich plasma; GAGs, glycosaminoglycans; GF, growth factor; hNPCs, human nucleus pulposus cells; ICA, icariin; ITS, insulin transferrin selenium; KOOS, knee injury and osteoarthritis outcome score; NO, nitric oxide; OA, osteoarthritis; PEG, polyethylene glycol; PRP, platelet-rich plasma.

In summary, the equivalence of FD-PRP to fresh-frozen PRP has been reported in the context of bone, muscle, tendon and cartilage research; the clinical efficacy of FD-PRP is endorsed by a preliminary study in knee osteoarthritis [49]. Lyophilized PRP has been combined successfully with alginate and is comparable to fresh-frozen PRP; this combination product is promising in bone fusion with relevant advantages in dosing and precise standardization. Other studies endorse the applicability of FD-PRP in the creation of different combination products for bone–tendon healing or volumetric muscle loss [59]. 

## 4. Dentistry Research

The use of PRP in oral implantology dates back to the 1990s [61], initially aiming to stimulate alveolar bone regeneration to help achieve faster dental implant stabilization. Two different formulations were very popular in dentistry: PRP (prepared from anticoagulated blood) and PRF (prepared from blood) [62] (see Figure 2). Their influence in periodontal ligament and alveolar bone regeneration has been the main focus of clinical and experimental research during recent decades. In this way, to leverage the potential of lyophilized plasma products in implantology, FD-PRF was proven to enhance the expression of transcription factors related to alveolar bone regeneration in mice compared to traditional PRF [63] (Table 3). Moreover, the equivalence between fresh-frozen PRP and FD-PRP was shown in terms of PDGF-BB, TGF-β and VEGF concentrations and stimulation of bone formation in immunocompromised mice [64]. However, in an apparent contradiction, rabbit BMSCs differentiation and proliferation was better in CaCl_2_-activated fresh PRP compared with FD-PRP [65].

In another example, FD-PRP was used to enhance the biological activities of fresh PRP formulations. Supplementing fresh PRF with allogeneic FD-PRF increased the concentration of GFs and other signaling cytokines, thereby enhancing bone regeneration in two different rodent calvarial models [66,67].

To fulfill the needs of bone formation and remodeling over time, a sustained release of GFs is desirable. In this way, the combination of FD-PRP with alginate and chitosan helped to slow down the release of TGF-β, PDGF, IGF, VEGF and TSP-1 [68].

Oral soft tissues have a faster turnover than the alveolar bone. Thus, isolating both compartments using membranes for guided bone regeneration overcomes obstacles caused by the different kinetics of tissue formation. Hence, PRF is investigated as an option for periodontal regeneration. A proprietary closed system, which prepared PRF, showed that membrane freeze-drying was not an obstacle for cell adhesion and proliferation [69]. Accordingly, a randomized clinical trial, comparing FD-PRF vs. fresh PRF in guided bone regeneration surgery did not find any difference in their clinical performance [70]. An optimized membrane, in terms of biological activities and mechanical properties, was elaborated with FD-PRF/chitosan/collagen [71] to be used in periodontal disease. A biodegradable polymer mesh coated with FD-PRP was effective in promoting periodontal ligament cell adhesion and growth as well as stimulating angiogenic mechanisms [72].

**Table 3:** Studies in dentistry (9).

Author, Year (Reference)	FD-PRP Based Product/Stability	Study Type/Cells/Animal Model	Results
Ansarizadeh M 2019 [71]	PRF (single spin): frozen (−80 °C) vs. FD Mixed with chitosan/collagen	FTIR, SEM, Young’s modulus, hMSCs viability, ALP activity, membrane degradation rate.	Optimized membrane composition based on experimental algorithms: Chitosan: collagen 4:1 + 0.58 mg/mL PRF Increased ALP activity (osteogenic differentiation) with PRF
Kardos D 2019 [69]	PRF (single spin) open vs. closed system: fresh, frozen (−20 °C), FD-PRF (−80 °C 30 min, −54 °C o/n)	Tensile strength, surface microstructure, plasmin activity, MSC and human gingival fibroblasts adhesion and proliferation, pro-collagen synthesis	Lower tensile strength in fresh PRF; frozen and thawed PRF lower plasmin activity than fresh and FD-PRF. Improved MSC adhesion in frozen and FD-PRF, no differences in gingival fibroblasts, no differences in pro-collagen synthesis
Li J 2017 [66]	PRP (double spin), vs. FD-PRP/PCL vs. traditional PRP (thrombin/Ca^2+^-activated)/PCL vs. PCL	DPSCs: migration, proliferation, ALP activity, osteogenic genes expression (RUNX2, OCN, OPN) In vivo rat calvarial defect assesment	FD-PRP/PCL better than traditional PRP/PCL and PCL, in terms of osteogenesis (RUNX2, OCN, OPN) and mineralization Faster rate of in vivo bone formation with FD-PRP/PCL
Li Q 2014 [63]	FD-PRF vs. traditional PRF (porcine)	ABs, PDLs and DFs: proliferation, migration, differentiation/mineralization, steogenic genes expression (RUNX2, MGP) In vivo, nude mice, calvarial defect: histology—bone formation, collagen synthesis; CT scans—bone regeneration	FD-PRF promotes RUNX2 expression in alveolar bone, not in dental follicle, partially in periodontal progenitors Histology reveals enhanced bone formation with FD-PRP (nodules after 14d) compared with fresh PRF
Liu Z 2019 [67]	FD-PRF vs. FD-PRF supplementing fresh PRF vs. fresh PRF (prepared from New Zealand rabbits)	PDGF-AB, TGF-b1 and VEGF quantification SEM hBMMSCs: proliferation (MTT), differentiation, mineralization nodules In vivo rabbit calvarial defect: histomorphometric analyses, CT scan	Sustained factor release in fresh+FD-PRF No differences in hBMMSCs proliferation Higher differentiation characteristics in FD-PRF Higher bone formation area at 12 weeks in fresh PRF, FD-PRF group, fresh+FD-PRF FD-PRF maintains the ability to promote bone proliferation and chemotaxis in osteoblasts
Nakatani Y 2016 [64]	FD-PRP vs. fresh PRP	PDGF-BB, TGF-b and VEGF release immunocompromised mice BULB: Bone formation histology and immunohistochemistry	Equivalent GFs release in fresh vs. FD-PRP Maintained bone regeneration at 4 and 8 weeks
Wang L 2019 [68]	(FD-PRP vs. fresh PRP) mixed with chitosan and alginate	TGF-b1, PDGF-AB, IGF-1, VEGF and TSP-1 release during 28d MC3T3-E1 murine osteoblast precursor cell line: Cytotoxicity, proliferation mineralization, osteogenic gene expression (OPN, OPG, Runx2, bone sialoprotein, osteocalcin)	More rapid GF release from FD-PRP composites versus sustained release from PRP composites Better osteogenic performance in FD-PRP in early stages Better osteogenic mineralization in fresh PRP at later stages
Xie Y 2020 [65]	CaCl_2_-activated fresh PRP vs. FD-PRP	PDGF-AB, TGF-b and VEGF quantification SEM Rabbit BMMSC: proliferation and differentiation (ALP activity, OCN, BMP-2 gene expression)	Higher PDGF, TGF and VEGF release in fresh PRP Enhanced osteogenic differentiation with fresh PRP at 1, 3, 6 and 9 days.
Zhang J 2017 [70]	Autologous fresh PRF (single spin) vs. autologous FD-PRF	Randomized clinical trial in guided bone regeneration (alveolar bone). Healing mucosa score (color, shape and quality), clinical outcomes (pain, color, swelling) at 24h, 3 and 7 days; computed tomography at 4 months	No statistical differences in soft-tissue healing or bone formation. No bone infection. Similar ratios of bone and soft connective tissues in the histological sections

Abbreviatures: AB, alveolar bone osteoblasts; ALP, alkaline phosphatase activity; BMMSCs, bone marrow-derived mesenchymal stem cells; DF, dental follicle progenitors; DPSCs, dental pulp stem cells; FD, freeze-dried; FD-PRF, freeze-dried platelet-rich fibrin; FD-PRP, freeze-dried platelet-rich plasma; FTIR, fourier transform infrared; hBMMSCs, human bone marrow-derived mesenchymal stem cells; hMSCs, human mesenchymal stem cells; MC3T3-E1, SEM, scanning electron microscopy; PCL, polycaprolactone; PDL, periodontal ligament fibroblasts; PRF, platelet-rich fibrin; PRP, platelet-rich plasma.

In summary, FD-PRP is valuable for promoting alveolar bone regeneration either when used alone or when added to fresh PRP to augment GF concentrations. Moreover, the controlled release of GF and the creation of membranes for guided bone regeneration can be attained when FD-PRP is mixed with biomaterials.

## 5. The Way Forward

PRP therapies are best known for their benefit in wound healing, osteoarthritis and alveolar bone repair. However, substantial challenges remain to be addressed before PRPs evolve from off-label use to on-label prescription and their costs reimbursed by health insurance. The unanimously identified obstacle, hindering advancement in PRP science, is the lack of standardization in PRP formulations, a challenge that could be met somewhat by lyophilization.

As shown in the present review, freeze-drying preserves platelet function, cytokine concentration and their function. The easiness of combining PRP with biomaterials is guaranteed, and combination products can be designed to control the kinetics of cytokine release and match more specifically the needs of the target tissue. In addition, FD-PRP is promising to functionalize bioprinted constructs [73]).

Implementing efficient freeze-drying procedures for allogeneic platelet-rich plasma formulations are multipurpose, first, guiding scientific investigations, and second leveraging treatment feasibility (Table 4). Actually, the platelet number and the levels of relevant cytokines can be accurately determined, which can inform a research hypothesis about the mechanism of action. Furthermore, product standardization facilitates conducting homogeneous clinical trials informative enough to establish the superiority (or not) of PRP over other treatments. Another fundamental research question is to clarify the biological advantages of PCs over lysates or releasates, i.e., preserving platelet integrity over using platelet’s secretome, in the different clinical conditions.

On the other hand, expediency is guaranteed, as lyophilized products reduce the need for specific facilities and specialized staff and are readily available on demand.

If we improve our understanding of PRP assets, selecting donors among healthy people might be grounded in the identification of core PRP elements related to clinical efficacy, e.g., application of young PRP in tissues of older patients, or considering the influence of exercise/sport practice on PRP quality.

Freeze-dried allogeneic PRP has to be declared fit as per national guidelines. Grafting FD-PRP across blood groups may need specific guidelines regarding the titration of antibodies but the fact that FD-PRP requirements for regenerative medicine may differ from transfusion medicine needs to be considered.

The manufacturing of freeze-dried allogeneic PRP or PRF has to comply with the specific requirements overseen by the competent regulatory body in each country, in terms of manufacturing establishments and donor suitability. Of note is the presence of blood-type immunoglobulin M antibodies, i.e., specific antiviral IgM.

Preferably, FD-PRP should be used after titration of antibodies, limiting the level of IgM ABO antibody titers.

## Figures and Tables

**Table 4:** Advantages and weaknesses of freeze-dried platelet-rich plasma.

Advantage	Weaknesses
Preserve PRP bioactivity Allows standardization of platelet number and growth factor levels, doses can be adjusted Avoids interdonor variability (if allogeneic) Easy to handle and mix with other biomaterials Quick reconstitution process by rehydration at the point of care, no need of specific equipment Stability at room temperature for several months No need of venipuncture at the point of care Saves time of preparation Immediate availability (off-the-shelf product) Timely use in case of emergency Ease of shipping and transport	Costs of the research needed to fulfill regulatory requirements Costs of fabrication Minor risks of contamination and disease transmission Needs optimization and standardization of freeze-drying procedures
